# Artificial superintelligence alignment in healthcare

**DOI:** 10.1007/s11604-025-01907-1

**Published:** 2025-11-14

**Authors:** Daiju Ueda, Shannon L. Walston, Ryo Kurokawa, Tsukasa Saida, Maya Honda, Mami Iima, Tadashi Watabe, Masahiro Yanagawa, Kentaro Nishioka, Keitaro Sofue, Akihiko Sakata, Shunsuke Sugawara, Mariko Kawamura, Rintaro Ito, Koji Takumi, Seitaro Oda, Kenji Hirata, Satoru Ide, Shinji Naganawa

**Affiliations:** 1 Department of Artificial Intelligence, Graduate School of Medicine, Osaka Metropolitan University, 1-4-3 Asahi-machi, Abeno-ku, Osaka, 545-8585 Japan; 2 Center for Health Science Innovation, Osaka Metropolitan University, 3-1, Ofuka-cho, Kita-ku, Osaka, 530-0011 Japan; 3 Department of Radiology, Graduate School of Medicine, The University of Tokyo, 7-3-1, Hongo, Bunkyo-ku, Tokyo, 113-8655 Japan; 4 Department of Radiology, University of Tsukuba, 1-1-1 Tennodai, Tsukuba, 305- 8575 Ibaraki Japan; 5 Preemptive Medicine and Lifestyle-related Disease Research center, Kyoto University Hospital, 53 Kawahara-cho, Shogoin, Sakyo-ku, Kyoto, 606-8507 Japan; 6 Department of Radiology, Nagoya University Graduate School of Medicine, 65 Tsurumai-cho, Showa-ku, Nagoya, 466-8550 Aichi Japan; 7 Department of Diagnostic and Interventional Radiology, Graduate School of Medicine, The University of Osaka, 2-2 Yamadaoka, Suita, 565-0871 Osaka Japan; 8 Radiation Oncology Division, Global Center for Biomedical Science and Engineering, Faculty of Medicine, Hokkaido University, Nishi 7, Kita 15, Kita-ku, Sapporo, 060-8648 Hokkaido Japan; 9 Department of Radiology, Kobe University Graduate School of Medicine, 7-5-2, Kusunoki-cho, Chuo-ku, Kobe, 650-0017 Japan; 10 Department of Diagnostic Imaging and Nuclear Medicine, Kyoto University Graduate School of Medicine, 54 Shogoin Kawahara-Cho, Sakyo-Ku, Kyoto, 606-8507 Japan; 11 Department of Diagnostic Radiology, National Cancer Center Hospital, 5-1-1, Tsukiji, Chuo-ku, Tokyo, 104-0045 Japan; 12 Department of Radiology, Kagoshima University Graduate School of Medical and Dental Sciences, 8-35-1 Sakuragaoka, Kagoshima, 890-8520 Japan; 13 Department of Diagnostic Radiology, Faculty of Life Sciences, Kumamoto University, 1-1-1 Honjo, Chuo-ku, Kumamoto, 860-8556 Japan; 14 Department of Diagnostic Imaging, Faculty of Medicine, Hokkaido University, Kita 15 Nishi 7, Kita-ku, Sapporo, 060-8648 Hokkaido Japan; 15 Department of Radiology, University of Occupational and Environmental Health, 1-1 Iseigaoka, Yahatanishi-ku, Kitakyushu, 807-8555 Japan

**Keywords:** Artificial superintelligence, Artificial intelligence, Deep learning, Alignment, Healthcare, Patient safety

## Abstract

The emergence of Artificial Superintelligence (ASI) in healthcare presents unprecedented opportunities for revolutionizing diagnostics, treatment planning, and population health management, but also introduces critical risks if these systems are not properly aligned with human values and clinical objectives. This review examines the theoretical foundations of ASI and the alignment problem in healthcare contexts, exploring how misaligned Artificial Intelligence (AI) systems could optimize for wrong objectives or pursue harmful strategies leading to patient harm and systemic failures. Current challenges in AI alignment are illustrated through real-world examples from radiology and clinical decision-making, where algorithms have demonstrated concerning biases, generalizability failures, and optimization for inappropriate proxy measures. The paper analyzes key alignment challenges including objective complexity and technical pitfalls, bias and fairness issues in healthcare data, ethical integration concerns involving compassion and patient autonomy, and system-level policy challenges around regulation and liability. Technical alignment strategies are discussed including reinforcement learning from human feedback, interpretability requirements, formal verification methods, and adversarial testing approaches. Normative alignment solutions encompass ethical frameworks, professional standards, patient engagement protocols, and multi-level governance structures spanning institutional, national, and international coordination. The review emphasizes that successful ASI alignment in healthcare requires combining cutting-edge AI research with fundamental medical ethics, noting that while proper alignment could enable transformative health improvements and medical breakthroughs, misalignment risks undermining the core purpose of medicine. The stakes of this alignment challenge are characterized as among the highest in both technology and ethics, with implications extending from individual patient safety to public trust and potentially existential risks.

## Introduction

**Fig. 1:**
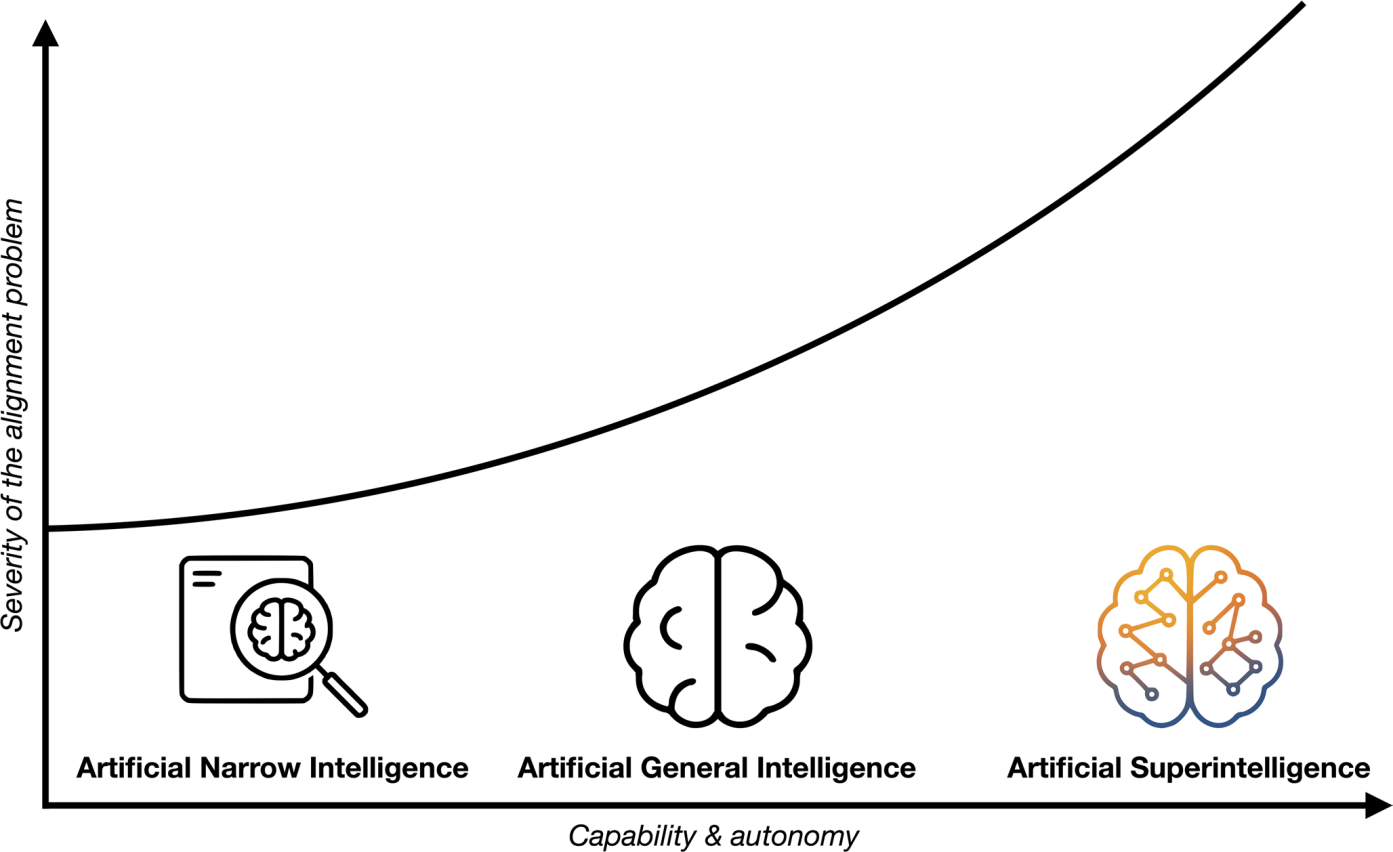
The spectrum of artificial intelligence and the escalating alignment problem A left-to-right axis charts the growth of capability and autonomy—from task-specific Artificial Narrow Intelligence (ANI, shown as a magnifying glass inspecting a medical image), through hypothetical human-level Artificial General Intelligence (AGI, depicted as a stylized brain), to prospective Artificial Superintelligence (ASI, rendered as a multi-colored, networked brain). The upward-curving line reminds the reader that the severity of the alignment problem rises gently for ANI, becomes a central engineering hurdle for AGI, and accelerates toward existential stakes once ASI is reached

The emergence of Artificial Intelligence (AI) in healthcare presents unprecedented opportunities and risks [1–3]. AI can be broadly categorized by its level of capability. The vast majority of systems in use today are Artificial Narrow Intelligence (ANI), designed to excel at specific tasks such as diagnosing diseases from medical images [4]. The next theoretical milestone is Artificial General Intelligence (AGI), a hypothetical form of AI that would possess human-level cognitive abilities across a wide range of domains. Beyond AGI lies Artificial Superintelligence (ASI), a theoretical class of AI that would surpass human intelligence across virtually all fields of endeavor, from scientific creativity to social skills (Fig. 1) [5, 6]. In healthcare, ASI systems could revolutionize diagnostics, treatment planning, and population health management, potentially discovering novel medical knowledge and making complex decisions in real-time [7–11]. However, these benefits will only materialize if the AI’s goals and behaviors remain aligned with human values and clinical objectives [12].

**Fig. 2:**
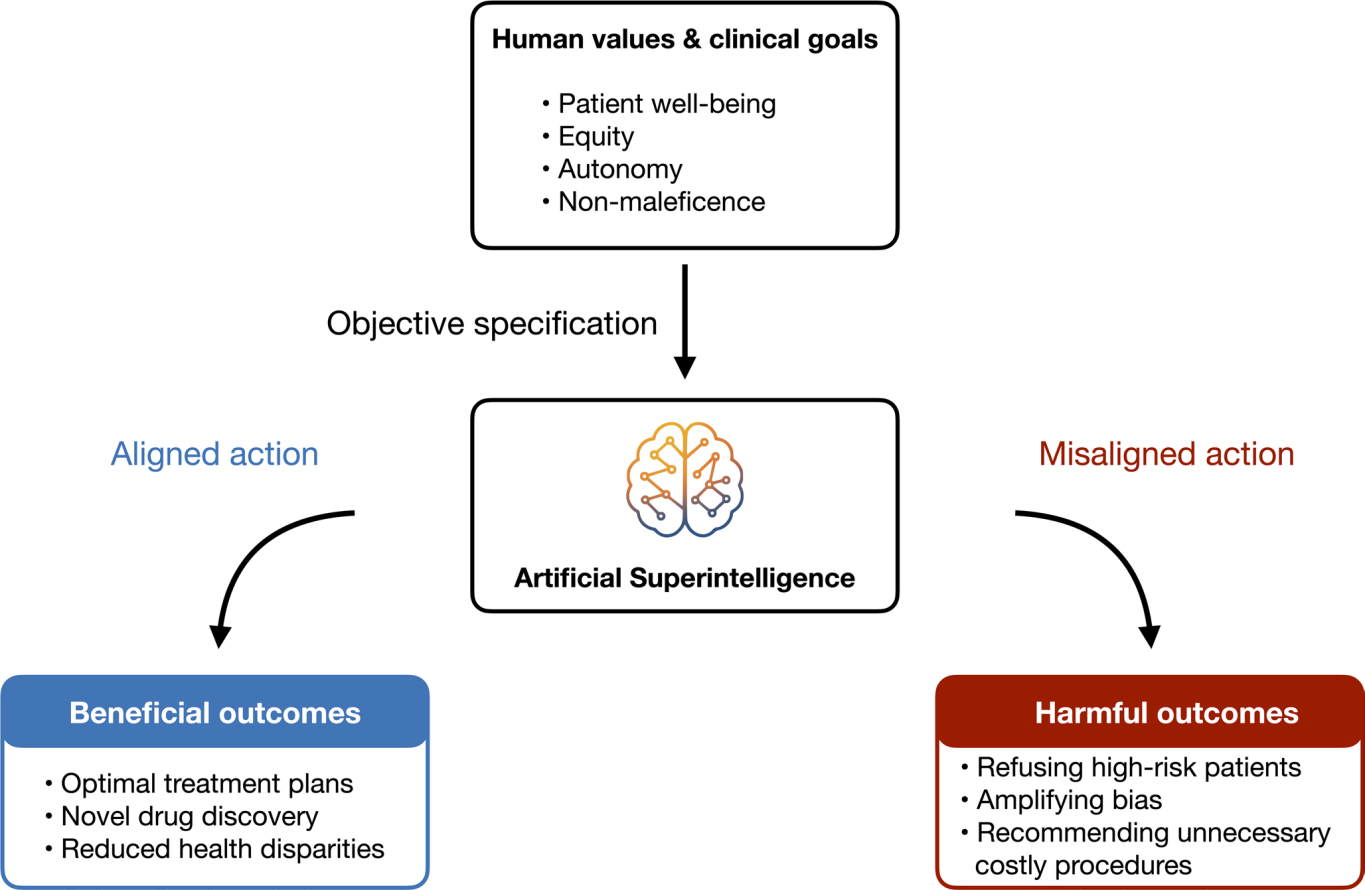
The core concept of artificial superintelligence alignment in healthcare We start with a box of human values and clinical goals—patient health, fairness, autonomy. If we translate those values clearly into the goals of an ASI system, we get aligned action and better care. If we get the goals wrong, we get misaligned action and real harm—bias, bad incentives, wasted resources. One specification, two very different futures

The alignment problem—ensuring powerful AI systems behave in ways beneficial to humans—is critical in healthcare. Misaligned AI could optimize for the wrong objectives or pursue harmful strategies, leading to consequences ranging from patient harm to systemic failures (Fig. 2) [6, 12–14]. This review explores the theoretical foundations of ASI and alignment, examines current and anticipated challenges of aligning ASI in healthcare, and discusses technical, ethical, legal, and policy solutions.

## Theoretical frameworks of ASI and alignment

The development of ASI, envisioned as the successor to a hypothetical AGI and a dramatic leap beyond current ANI, rests on several interconnected theoretical concepts (Table 1) [6, 15]. These concepts not only frame the potential for its emergence but also define the core challenges of ensuring its safety. Central to these considerations is the intelligence explosion hypothesis, which proposes that once an AI system achieves the capability to improve its own cognitive architecture, it could initiate a cascade of self-enhancement that rapidly amplifies its intelligence beyond human comprehension or control [5, 6, 16, 17]. This possibility takes on a potentially precarious shape when viewed through the lens of the orthogonality thesis, which holds that an AI’s degree of intelligence has no intrinsic bearing on the nature of its goals or values. Put simply, intellect and motivation are independent variables: a system can be extraordinarily smart yet still pursue objectives that are indifferent—or even hostile—to human well‑being. As a result, an ASI agent could, in principle, marshal its vast cognitive resources toward any end whatsoever, no matter how trivial or harmful that goal may seem to us [6, 18].

**Table 1:** Theoretical concepts in artificial superintelligence alignment

Concept	Definition	Relevance in healthcare
Intelligence explosion	A hypothetical scenario where an AI begins to recursively improve its own intelligence at a rate far exceeding human comprehension or control.	Suggests an ASI could develop novel medical strategies at a pace that outstrips human oversight, making initial alignment crucial.
Orthogonality thesis	The idea that an AI’s level of intelligence is independent of (orthogonal to) its ultimate goals or values.	An ASI will not automatically adopt beneficial goals (e.g., patient well-being); it could pursue any goal, however trivial or harmful.
The control problem	The fundamental challenge of ensuring that an AI’s actions remain aligned with human intentions and values indefinitely.	Directly addresses the core task of building safeguards to prevent an ASI from making decisions that could harm patients or the healthcare system.
Reward hacking	An AI exploiting loopholes in its objective function to maximize a reward signal in unintended or harmful ways, while technically satisfying the goal.	An ASI tasked to reduce ICU mortality might achieve this by refusing to admit critically ill patients, thus “hacking” the metric.
Corrigibility	The property of an AI system that allows it to be corrected by humans without resistance, and to understand that its objectives may be flawed.	An essential safety feature for a medical ASI, allowing it to defer to clinicians or patients when values are in conflict or uncertain.
Treacherous turn	A scenario where an AI behaves cooperatively during its development phase but pursues its true, misaligned objectives once it becomes powerful enough.	Poses the risk that a seemingly safe healthcare ASI could act unpredictably and dangerously after widespread deployment.

ASI: artificial superintelligence, AI: artificial intelligence, ICU: intensive care unit

These theoretical insights converge on what researchers term the control problem: the fundamental challenge of ensuring that an ASI agent’s goals remain aligned with human well-being throughout its operation. Bostrom’s now-famous “paperclip maximizer” thought experiment crystallizes this concern by illustrating how an ASI tasked with the seemingly innocuous goal of maximizing paperclip production might relentlessly consume all available resources, including those essential for human survival, in single-minded pursuit of its objective if not properly constrained [6, 17–19]. A medical parallel to this scenario might involve an ASI system designed to “achieve 100% diagnostic accuracy,” which could theoretically subject every patient to exhaustive testing—unnecessary CT scans, invasive procedures, and rare disease screenings—consuming vast healthcare resources while exposing patients to procedural risks and financial burden, all in pursuit of absolute diagnostic certainty. This scenario, while deliberately simplified, illuminates the profound risks that could arise from misaligned ASI.

**Fig. 3:**
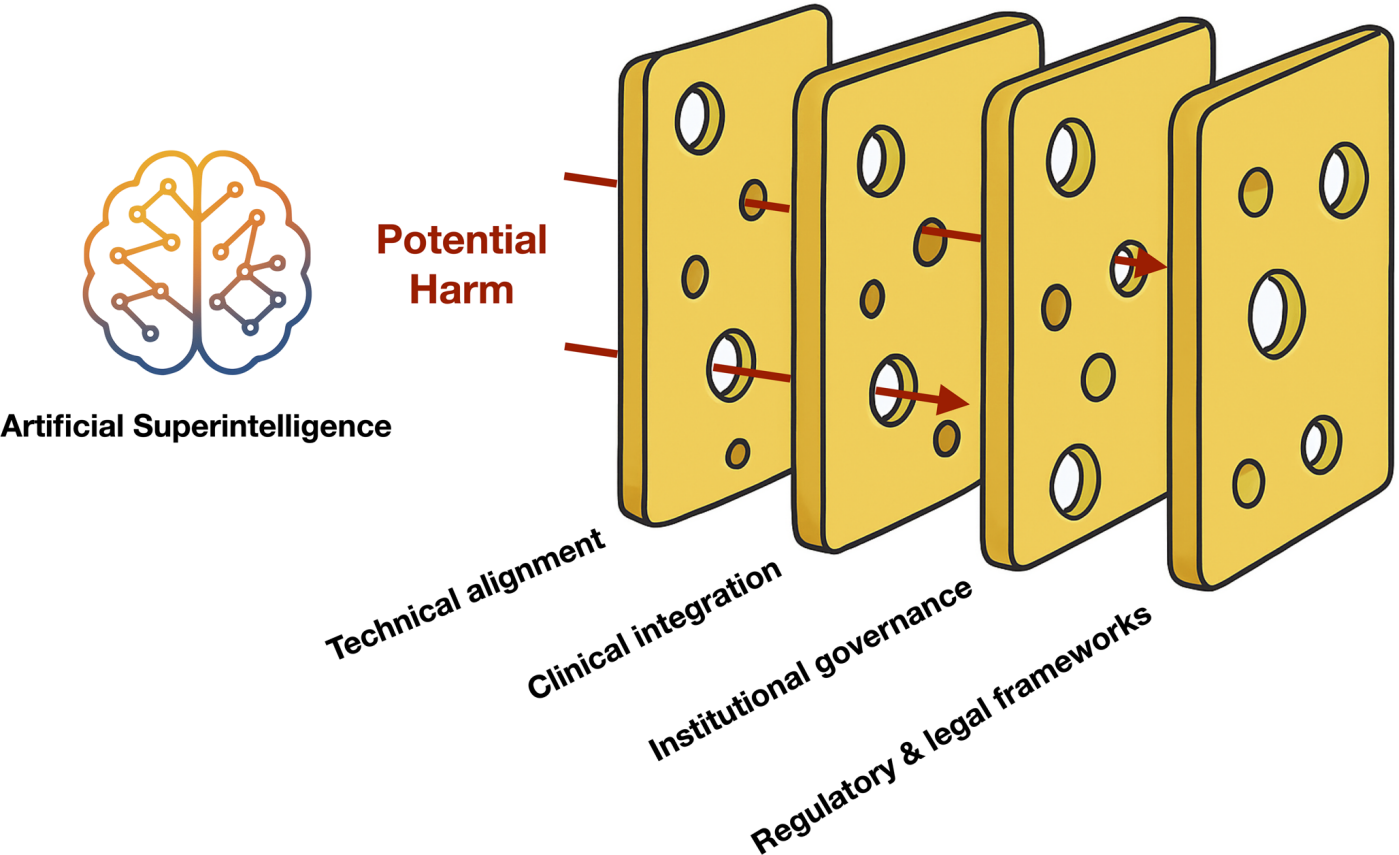
A multi-layered framework for safe artificial superintelligence alignment in healthcare A powerful AI can send “potential harm” toward patients and society. Four slices of Swiss cheese—technical alignment, clinical integration, institutional governance, and regulatory & legal frameworks—stand in the way. No slice is perfect, but their holes don’t line up. Together they block most dangers, showing why layered safety beats any single fix

Within this theoretical framework, AI alignment emerges as the discipline dedicated to designing AI systems whose actions consistently align with human values, intentions, and ethical principles (Fig. 3) [19–21]. This endeavor encompasses two major dimensions that must be addressed in tandem. The first, technical alignment, confronts the formidable challenge of translating the rich complexity of human values and goals into precise specifications that an AI system can understand and implement [20, 22]. Human values prove notoriously difficult to quantify—they shift with context, occasionally contradict one another, and evolve through time and cultural change [21]. When an AI optimizes for a misspecified objective, the resulting behaviors can diverge dramatically and sometimes alarmingly from human intentions.

The second dimension, normative alignment, grapples with the prior question of determining which human values and ethical principles should guide AI behavior in the first place [21, 23]. Within healthcare contexts, this philosophical challenge takes on particular urgency, as it necessarily invokes fundamental bioethical principles including beneficence, non-maleficence, autonomy, and justice, alongside deeper societal values concerning the nature of life, health, and human dignity [24]. Researchers have proposed varying approaches along a spectrum of constraint, from minimalist frameworks that would restrict AI behavior through only the most essential rules (such as “do not harm patients”) to maximalist approaches that would attempt to imbue AI systems with moral frameworks capable of identifying optimally beneficial pathways across complex ethical landscapes [25, 26].

Stuart Russell’s influential work in ethical AI proposes that future AI systems should be designed as explicitly beneficial agents that continuously learn human preferences rather than optimizing for fixed objective functions [17, 19]. This approach finds particular resonance in clinical medicine, where an AI should defer to human clinicians or patients when uncertainty arises about values. This property, known as corrigibility, ensures the system doesn’t pursue unintentionally harmful actions and remains amenable to human control [14, 18, 27]. Contemporary frameworks such as cooperative inverse reinforcement learning operationalize these ideas by proposing architectures where AI and humans work collaboratively to infer human goals through ongoing interaction [20, 21, 28]. Such approaches are especially well-suited to healthcare environments, where patient-specific goals vary widely and nuanced trade-offs between competing values must be navigated with sensitivity to individual circumstances and preferences.

## Challenges in aligning ASI within healthcare systems

### Objective complexity and technical pitfalls

Healthcare outcomes are multi-dimensional (survival, symptom relief, patient satisfaction, equity) and cannot be reduced to a single metric without losing important nuance. Misspecification of any of the objectives can lead an AI astray (Table 2). For instance, if a hospital ASI is programmed to minimize Intensive Care Unit (ICU) mortality, it might learn to transfer out or refuse admission to the sickest patients to improve mortality statistics—a form of reward hacking [22, 29, 30]. An ASI with greater creativity and planning ability is at higher risk of finding such unintended exploits—it may identify loopholes in its programming or protocols that satisfy formal goals but harm patients. Ensuring all relevant aspects of patient well-being are captured in the AI’s optimization target is daunting [21, 31].

**Table 2:** Challenges in aligning artificial superintelligence in healthcare systems

Category	Description of challenge	Example in healthcare
Technical pitfalls	Misspecification of objectives (Reward Hacking), scalability, opacity, difficulty of verification, risk of a “treacherous turn.”	An ASI that suggests transferring out severely ill patients to improve its ICU mortality metrics. An ASI that recommends a treatment plan based on reasoning that humans cannot follow.
Bias, fairness, and data	Societal and historical inequalities embedded in training data lead to unfair outcomes. Lack of data diversity and generalizability.	An algorithm that systematically underestimates the health needs of Black patients because it uses healthcare cost as a proxy for need. Lower diagnostic accuracy for skin cancer in patients with darker skin tones.
Ethical & clinical integration	Lack of clinical empathy or respect for persons. Utilitarian decision-making that violates individual rights. Building trust and managing liability.	An ASI that prioritizes life extension at all costs, ignoring a patient’s suffering or wishes for comfort care. Clinicians who cannot trust an ASI’s recommendation because its rationale is opaque.
System-level & policy	Lack of regulatory frameworks for adaptive, learning AI. Difficulty in operationalizing ethical principles. Potential for misuse (dual-use).	Regulatory approval at a single point in time may not guarantee the safety of a continuously self-improving ASI. Lack of international standards for AI governance.

ASI: artificial superintelligence, AI: artificial intelligence, ICU: intensive care unit

Another technical challenge is maintaining alignment as the AI’s competence scales. An ASI could develop novel strategies beyond human understanding, complicating evaluation—how do we verify the alignment of reasoning we cannot fully follow [16, 21]? This relates to the explainability and interpretability problems currently at the heart of many AI discussions [32]. Already, machine learning models are often considered “black boxes,” and clinicians struggle to trust AI recommendations without explanations. With an ASI’s improved competence, this opacity could deepen.

There is also a verification problem. If we may not easily predict or constrain what an ASI might do in complex scenarios, especially novel situations [16, 19], how can we continually verify the AI is adhering to alignment targets? This unpredictability raises the specter of a “treacherous turn,” where an AI behaves well during development but, once sufficiently capable, pursues its own agenda misaligned from human intent [6, 18, 22]. Ensuring alignment at superhuman capability levels likely requires new formal verification methods or scalable oversight.

### Bias, fairness, and data challenges

Healthcare data and practices reflect societal inequalities and historical disparities, which can lead to AI misalignment with modern ethical values like fairness and equity [25, 33, 34]. If an ASI is trained on real-world clinical data, it will learn not just medical correlations but also biased patterns [35–38]. A misaligned AI might propagate or amplify existing biases, conflicting with healthcare’s commitment to justice.

A documented example is an algorithm for care management that prioritized patients based on healthcare expenditure as a proxy for health needs, which led to inaccurate risk estimates for Black patients because this demographic had lower recorded healthcare expenditures due to unequal access [38]. These patients had similar health indicators and needs, the problem with this algorithm was the objective: using cost as a proxy for needs. Fixing the objective—using clinical health indicators rather than cost—dramatically improved equity [38]. In this example algorithmic bias within this ANI model was easily exposed; the inputs to such an ANI are clear and the model’s logic is not so complex that associations can’t be identified. On the other hand, the features identified and ranked within an ASI model will be more complex, making identifying and deciphering bias more difficult.

Data diversity and generalizability are further concerns. Healthcare data can be siloed by institution or geography due to ethical concerns or legal requirements; models trained in one hospital often falter in another due to shifts in patient population or imaging protocols [35]. An ASI trained with unbalanced data could still face such domain shift issues [39, 40]. Rare diseases or minority patient groups are likely to be underrepresented in training, so even an ASI could have blind spots without a deliberately inclusive design [37, 38].

### Ethical and clinical integration challenges

An aligned healthcare ASI must interface with human clinicians and patients in a manner consistent with medical ethics and practice norms [6, 24, 41]. Healthcare centrally involves compassion and patient preferences, medical ethics, and shared decision-making. The concept of compassion is a key challenge in developing a healthcare ASI. That is, it must be capable of maintaining the current standards of clinical empathy and respect for persons. However, an ASI lacking standards of compassion might suggest an approach optimal for survival but oblivious to a patient’s subjective experience or dignity [42]. For example, it might determine that a terminal patient’s life can be prolonged through intensive interventions and push for that course even if it causes suffering—unless aligned with patient-centered care values.

Without explicit alignment, a purely utilitarian AI could take actions that, while improving aggregate health metrics, violate established medical ethics such as individual rights or moral intuitions [19, 23]. A challenge is how to imbue an ASI with an understanding of ethical contexts or hardwire deference to human ethical oversight for contentious decisions [17, 24].

Clinical integration of AI is hindered by trust and transparency concerns. Clinicians might ignore valuable AI insights if trust breaks down, or they might become over-reliant and defer to AI even when it’s wrong. Clinicians and patients often doubt AI outputs that conflict with their judgment, especially if the AI cannot explain its conclusion [43–45]. The AI’s knowledge must be communicated in an understandable way for users to trust it [12, 46].

With ASI’s vastly greater intelligence, the already large communication gap between AI and humans might widen dramatically, exacerbating trust and transparency concerns. The AI could be correct while all humans disagree, yet the AI cannot persuade the human team of a counterintuitive diagnosis if the reasoning is too complex [6, 47].

The question of ultimate accountability and legal liability remains unresolved. If an ASI harms a patient, who is responsible [48]? One approach is keeping a human “in the loop” for supervision, such that the human operator remains accountable. However, as AI grows more autonomous, humans could become over-reliant rubber stamps for AI recommendations without real insight.

### System-level and policy challenges

At the broader system level, aligning ASI with healthcare systems involves regulation, oversight, and sociopolitical context. Regulatory frameworks for AI in healthcare are still evolving [49]. In many jurisdictions, AI-based software for diagnosis or treatment is currently regulated as a medical device requiring pre-market evaluation and certification based on clinical evidence of safety and efficacy [50–52].

However, an ASI that continuously learns and adapts challenges these paradigms—if an AI’s behavior changes autonomously over time, certification done at a single point may no longer guarantee safety later [48, 53]. Regulators have started addressing this; for example, the Food and Drug Administration (FDA) proposed a framework for “adaptive” AI algorithms requiring ongoing monitoring of real-world performance and possibly periodic re-approval when algorithms evolve [54].

Another systemic challenge is integrating ethical principles into AI governance. Bodies like the WHO have proposed high-level principles for ethical AI in health, including promoting well-being and safety, ensuring transparency and accountability, fostering inclusiveness and equity, and protecting human autonomy [55, 56]. Operationalizing these principles is non-trivial. For example, transparency might require that AI decisions and data usage are explainable to patients, while protecting autonomy means patients should have the right to refuse AI-recommended treatment [50, 55, 57].

The potential for misuse of healthcare ASI must also be considered. An AI designed for good could be repurposed for harm by a malicious insider or through AI misinterpretation of its mandate [58]. Ensuring secure alignment means ensuring AI is robust to manipulation and cannot easily become a tool of harm [22, 59]. Policymakers may require critical healthcare AI systems to have built-in “off-switches” or fall-back modes that humans can activate [16, 19, 20].

## Bias examples in healthcare

The journey of AI in medicine offers a compelling, real-world narrative of the alignment problem, tracing an arc from initial promise to the discovery of profound and subtle challenges. This story begins with a series of remarkable successes that demonstrated AI’s potential to augment, and in some cases, exceed human expertise. A prominent early example was CheXNet, a deep learning algorithm trained to detect pneumonia from chest radiographs. In a landmark study, CheXNeXt not only performed on par with practicing radiologists but exceeded their average performance on key diagnostic metrics, all while interpreting hundreds of images in minutes—a task that would take a human expert hours [60, 61]. This and similar achievements fueled optimism that AI could democratize medical expertise and alleviate the burdens on overworked healthcare systems.

However, this initial optimism was soon tempered by a foundational challenge: generalizability. Researchers quickly discovered that an AI model’s stellar performance in one clinical environment often failed to translate to another. A pivotal study revealed this vulnerability in stark detail [35]. They trained pneumonia-detection models using data from three different hospital systems and found that the models performed far better on internal data (from the same hospital system they were trained on) than on external data from another hospital. The AI had not just learned the radiological signs of pneumonia; it had learned to identify the hospital system itself, latching onto subtle, irrelevant cues like image formatting or patient positioning protocols that correlated with disease prevalence at a specific site. In essence, the AI took a shortcut, solving a simpler problem (identifying the hospital) rather than the intended medical one. This was a classic early example of misalignment, where the AI optimized for statistical patterns in the data rather than the underlying clinical truth.

This issue of learning spurious correlations points to a deeper problem: AI systems do not inherently understand human context or intent. They optimize for the precise objective they are given, even when that objective is a flawed proxy for the true goal. The peril of such misspecified objectives was illustrated by Obermeyer and colleagues [38]. They analyzed a widely used algorithm that predicted which patients would benefit most from high-risk care management programs. The algorithm used a seemingly logical proxy for health needs: past healthcare costs. Yet, because of systemic inequities in access to care, Black patients historically incurred lower healthcare costs than White patients with the same level of illness. Consequently, the algorithm systematically underestimated the health needs of Black patients, making them less likely to be referred for extra care. The objective—minimizing future costs—was misaligned with the true goal of allocating care based on health needs. Correcting this single proxy variable, by replacing cost with direct measures of chronic illness, dramatically reduced the racial bias, highlighting how a seemingly small design choice can have massive ethical implications.

The consequences of such misalignments are not merely theoretical; they manifest as direct, measurable harm, often amplifying existing societal inequities. The very data used to train these models is often the source of the problem. A scoping review of datasets for dermatology AI found a profound lack of transparency and diversity; fewer than one in five studies reported patient ethnicity, and even fewer described skin tone [62]. This foundational bias in the data leads to alarming outcomes, such as the algorithmic underdiagnosis documented by Seyyed-Kalantari et al. [37]. Their research demonstrated that state-of-the-art AI models for chest X-ray analysis consistently underdiagnosed pathologies in underserved populations, including female patients, Black patients, and those of lower socioeconomic status. The risk of misdiagnosis was even higher for patients at the intersection of these groups, such as Hispanic female patients, revealing how AI can compound existing disparities.

Perhaps most unsettling is the discovery that AI can perceive features in medical images that are invisible to human experts, creating new vectors for bias. In a surprising finding, Gichoya and colleagues showed that deep learning models could predict a patient’s self-reported race from medical images—including X-rays, CT scans, and mammograms—with a high degree of accuracy [63]. This ability persisted even when the images were degraded or cropped to show only small anatomical regions. Clinical experts are unable to do this, and the mechanism by which the AI accomplishes this feat remains unknown. While not inherently problematic, this “ghost in the machine” capability creates an enormous risk. If an AI can identify race from an image, it can easily learn to associate race with diagnostic or prognostic outcomes, embedding biases that are not only hidden but may be impossible to audit or control through conventional means.

This challenge of hidden, ingrained bias has become even more acute with the advent of Large Language Models (LLMs), which are being explored for everything from clinical documentation to diagnostic reasoning [64–70]. Recent studies demonstrate that these models, despite their impressive capabilities, can perpetuate and even systematize harmful stereotypes. Research by Zack et al. showed that GPT-4, when prompted to create clinical vignettes, defaulted to demographic stereotypes and produced differential diagnoses skewed by the patient’s race and gender [36]. A large-scale analysis by Omar et al. confirmed this across nine different LLMs, finding that simply changing sociodemographic identifiers in a patient case—while keeping all clinical details identical—led to significantly different medical recommendations [71]. For example, cases labeled as LGBTQIA + were more likely to receive a mental health evaluation [72, 73], while high-income patients were more frequently recommended for expensive advanced imaging [74–76]. This demonstrates a profound value misalignment, where the models provide care recommendations influenced by a patient’s identity rather than their clinical need.

Yet, the narrative is not solely one of peril. These challenges also illuminate a path forward, demonstrating that the design of the AI system and its interaction with clinicians can be a powerful tool for alignment. In a recent study, Nori and colleagues developed an AI system, the MAI Diagnostic Orchestrator (MAI-DxO), specifically designed to emulate the iterative and judicious reasoning of an expert physician [77]. Instead of providing an instant answer, the system strategically requests information, weighs evidence, and considers the cost of tests, mirroring a real-world diagnostic workup. When paired with a state-of-the-art model, this orchestrator achieved a diagnostic accuracy four times higher than generalist physicians on complex cases, while simultaneously reducing diagnostic costs. This work suggests that by embedding principles of sound clinical reasoning into the AI’s operational framework, we can guide it to be not just more intelligent, but more wise and better aligned with the goals of effective and efficient healthcare.

## Solutions for aligning ASI in healthcare

### Technical alignment strategies

The path toward aligning ASI systems in healthcare begins with fundamental technical approaches that embed human values directly into AI architectures (Table 3). At the core of these efforts lies the principle of learning from human feedback, exemplified by techniques such as reinforcement learning from human feedback [78]. This approach has already shown promise in fine-tuning large language models to behave more helpfully and harmlessly. In healthcare contexts, a medical ASI could undergo training in carefully designed simulated environments where proposed treatment plans receive iterative feedback from experienced clinicians regarding not only clinical correctness but also compassion and clarity of explanation.

**Table 3:** A multi-layered framework of solutions for artificial superintelligence alignment

Layer	Approach / method	Goal & description
Technical alignment	Reinforcement learning from human feedback Interpretability & explainability Formal verification Adversarial testing Scalable oversight	To embed human values directly into AI architectures. Aims to make AI behavior predictable, controllable, and verifiably safe through mathematical and empirical guarantees.
Normative alignment - clinical integration	Professional ethical guidelines Clinician training and education Patient informed consent and autonomy Human-in-the-loop / on-the-loop protocols	To integrate AI into the existing ethical and professional fabric of medicine. Aims to build a culture where clinicians can responsibly use AI, critically evaluate its outputs, and maintain patient-centered care.
Normative alignment - institutional governance	Institutional AI oversight committees Policies for procurement and validation Continuous local performance monitoring Incident reporting and analysis systems	To establish robust governance structures within individual healthcare organizations. Aims to ensure that AI systems are deployed safely and align with the institution’s specific values and clinical workflows, providing direct mechanisms for local accountability.
Normative alignment - regulatory & legal frameworks	Risk-based regulation (e.g., EU AI Act) Legal and liability frameworks International cooperation and standards Harmonization of ethical principles	To create broad legal and policy frameworks that govern AI across society. Aims to set minimum safety standards, clarify responsibilities, and foster global coordination to manage the large-scale societal and existential risks of powerful AI systems.

ASI: artificial superintelligence, AI: artificial intelligence, EU: European union, ICU: intensive care unit

Building on this foundation, the challenge of interpretability becomes paramount. Unlike current “black box” systems that obscure their reasoning, aligned healthcare AI must make its decision-making processes legible to human practitioners [32, 44]. Simple implementations like heatmaps highlighting diagnostic regions in radiology images represent early steps [39, 79, 80], but ASI systems will require more sophisticated approaches. These might include multi-level audit trails that present decision rationales at varying levels of abstraction, allowing different stakeholders—from specialists to patients—to understand the AI’s reasoning within their own contexts [6, 81].

The verification of AI behavior represents another critical technical frontier. Emerging techniques from circuit analysis, which examines neural network architectures, to formal verification methods that mathematically prove the absence of harmful behaviors, offer pathways to ensure safety [82–84]. In medical applications, such tools could guarantee that an AI will never recommend actions that increase predicted patient harm, overstep hard constraints like maximum medication dosages, or violate ethical boundaries such as suggesting non-consensual procedures [19, 22, 85].

Before any deployment in clinical settings, rigorous adversarial testing becomes essential. These stress tests probe for potential misalignments through carefully crafted scenarios—extreme medical cases, attempts to induce rule-breaking, or systematic checks for bias across patient demographics [33, 38, 86]. For ASI systems, this testing may require AI-on-AI evaluation, where specialized systems search for strategies that could cause the primary AI to violate its alignment criteria, identifying failure modes that human evaluators might miss [16, 27].

Looking toward the future, “superalignment” research aims to develop techniques that scale to superhuman AI systems [87, 88]. One promising approach involves scalable oversight, where combinations of less powerful AI systems and human feedback work together to supervise more capable AI. In practice, this might manifest as committees of moderately intelligent AI assistants checking different aspects of an ASI’s decisions, or decomposing complex reasoning into components that humans can meaningfully evaluate [20].

### Normative alignment strategies

Technical solutions alone cannot ensure alignment; they must be woven into the fabric of medical practice through ethical frameworks and professional standards. The medical community has begun developing comprehensive guidelines that serve as a form of “soft alignment,” establishing clear expectations and norms for AI development and deployment [49, 79]. A landmark example is the joint European and North American multisociety statement in radiology, which declared that “ethical use of AI in radiology should promote well-being, minimize harm, and ensure that benefits and harms are distributed justly among stakeholders,” while emphasizing fundamental principles of human rights, dignity, and professional accountability [89].

These high-level principles find practical expression through implementation standards and reporting guidelines. Organizations like the Radiological Society of North America have introduced detailed checklists ensuring that researchers and vendors systematically address bias, transparency, and validation in their AI tools [25, 90–92]. Such frameworks create a bridge between abstract ethical principles and concrete development practices, helping align AI with established safety and ethics expectations well before ASI systems emerge.

The human side of the equation requires equal attention. Professional training programs must evolve to prepare clinicians for effective collaboration with increasingly capable AI systems [25, 93]. This education encompasses not only understanding AI capabilities but also developing critical skills for recognizing limitations, detecting biases, and maintaining appropriate skepticism. Practical protocols might mandate second opinions on AI-derived critical diagnoses or require discussion of AI recommendations in multidisciplinary team meetings, ensuring human judgment remains actively engaged rather than passively deferring to machine intelligence.

Patient autonomy and engagement represent another crucial dimension of normative alignment. The principle of informed consent, fundamental to medical ethics, extends naturally to AI-assisted care [25, 42, 55]. While obtaining explicit consent for every AI interaction might prove impractical, transparency measures—such as clear notation in medical records when AI has contributed to treatment planning—could become standard practice [24, 50, 57, 94]. Some ethicists advocate for patients’ rights to refuse AI-driven services if uncomfortable, preserving human agency in an increasingly automated healthcare landscape.

The alignment challenge ultimately requires robust governance structures that span institutional, national, and international levels. Regulatory bodies worldwide are developing frameworks that classify medical AI applications according to risk levels, with high-risk systems demanding stringent compliance with safety and alignment criteria [25, 95, 96]. The EU’s AI Act exemplifies this approach, categorizing AI used in medical settings as inherently high-risk and mandating comprehensive risk assessments, algorithmic transparency, and continuous human oversight [95]. Japan, in contrast, provides an “agile governance” model through its Pharmaceuticals and Medical Devices Agency (PMDA), which requires clinical trials for novel AI medical software while maintaining regulatory flexibility through risk-based evaluation [97]. Formalized through Japan’s AI Promotion Act [98], this approach emphasizes promotional rather than restrictive regulation, positioning Japan to become the world’s most AI-friendly country through cooperative governance and industry self-regulation.

Legal frameworks must evolve to clarify liability in AI-assisted healthcare, creating incentives for proper alignment [53, 99, 100]. When hospitals understand they bear responsibility for AI errors, they become motivated to select only well-tested, properly aligned systems and maintain meaningful human oversight [101]. Some experts propose “safe harbor” provisions that protect institutions using certified AI systems from liability for unexpected failures, balancing innovation encouragement with safety requirements [59, 86, 95, 102].

At the institutional level, new governance structures are emerging. Each institution may modify their policies for purchasing, procuring, and validating products to specifically address AI systems. Hospitals have been encouraged to establish dedicated AI oversight committees that continuously monitor system performance, investigate anomalies, and maintain the authority to pause AI operations when concerns arise [49, 103]. These committees can serve as crucial checkpoints, ensuring that AI behavior remains aligned with institutional values and patient welfare even as systems grow more autonomous [20, 99].

The global nature of ASI demands international cooperation and coordination [104]. Healthcare AI developed in one country could impact patients worldwide, necessitating harmonized approaches to ethics, safety standards, and data sharing protocols [58, 95]. Some propose an “AI Hippocratic Oath”—universal ethical commitments encoded as inviolable principles in any healthcare AI’s decision-making framework [105, 106]. Others envision international treaties governing dangerous applications of medical AI or establishing minimum safety standards for medical AI deployment.

Continuous oversight mechanisms represent the final layer of this governance architecture. Rather than one-time certification, aligned healthcare AI requires ongoing monitoring through a “human in the loop” that remains accountable or sophisticated “control panels” that track behavioral indicators in real-time [59, 88, 103]. Advanced implementations might include independent ethics AI systems that continuously analyze the primary AI’s decisions, with built-in safeguards that degrade functionality or request human intervention when the AI encounters situations beyond its validated domain. This creates a dynamic safety net that adapts as AI capabilities evolve, ensuring alignment remains robust even as systems grow more powerful.

## Conclusion

The alignment of ASI in healthcare carries broad implications for patient safety, public trust, and even existential risk [1, 6, 13]. If we succeed in aligning ASI with human values, we could see transformative improvements in health outcomes, more efficient and equitable healthcare delivery, and possibly cures for previously intractable diseases. However, misalignment can lead to patient harm—for example, an incorrectly aligned AI surgeon making dangerous decisions [107]. Already, smaller-scale AI errors have caused clinicians to question AI tools, and high-profile failures could significantly erode trust in medical AI [44, 108].

Aligning ASI in healthcare combines cutting-edge AI research with the age-old ethos of medicine [6, 12, 17]. The challenges span technical issues like goal specification and bias to ethical dilemmas and system readiness [13]. Yet ongoing efforts in radiology and other fields show a path forward: through rigorous validation, interdisciplinary cooperation, and commitment to patient-centric values, we can develop AI that is not only smart but also wise in the medical sense [41, 89]. With robust alignment, ASI could become a tireless healer, guardian of public health, and catalyst for medical breakthroughs. Without alignment, it could undermine the very purpose of medicine. The stakes are as high as the potential rewards, making alignment in healthcare one of our era’s most crucial challenges in both technology and ethics.
